# 451. Pre- and Post-implementation Comparison of the Impact of Emergency Department (ED)-Based COVID-19 Point-of-Care Testing on ED Patient Metrics

**DOI:** 10.1093/ofid/ofad500.521

**Published:** 2023-11-27

**Authors:** Katherine Z J Fenstermacher, Eili Klein, Jeanne Mumford, Barbara Maliszewski, Gai Cole, Mustapha O Saheed, Richard E Rothman

**Affiliations:** Johns Hopkins University, Baltimore, Maryland; Johns Hopkins School of Medicine, Baltimore, Maryland; Johns Hopkins University, Baltimore, Maryland; Johns Hopkins University, Baltimore, Maryland; Johns Hopkins University, Baltimore, Maryland; Johns Hopkins, Baltimore, MD; Johns Hopkins University, Baltimore, Maryland

## Abstract

**Background:**

Use of Point of Care Testing (POCT) in Emergency Departments (EDs) may afford the opportunity to improve key ED operational metrics. We evaluated the impact of COVID-19 POCT on time to delivery of test results, rates of patients who left the ED with or without a COVID-19 diagnosis, and the amount of time COVID-19-negative patients spent in isolation.

**Methods:**

Retrospective pre-POCT (7/1/2020 – 8/31/2020, N=4105) and post-POCT (1/1/2021 – 3/31/2021, N=4795) implementation analysis of patients presenting to the Johns Hopkins Hospital Adult ED who received symptomatic COVID-19 testing. Both pre- and post-implementation POCT was performed using the Cepheid GeneXpert SARS-CoV-2 or SARS-CoV-2/Flu A/B/RSV nucleic acid tests, which have a run time of 45 minutes; pre-POCT occurred in the hospital central lab and POCT occurred in a POC testing lab established in the ED.

**Results:**

Post-implementation, the time from arrival to test result for all patients decreased by an average of 133.2 ± 3.3 minutes; the time to delivery of test result decreased by 177.4 ± 16.02 minutes for COVID+ patients. Reduction in time to test result was associated with a significant decrease in the percentage of patients who were discharged from the ED without their result being known prior to departure: pre-implementation, 8.7% of all tested patients left without a known result, with 21% of COVID+ patients leaving prior to receiving a diagnosis; post-implementation, only 0.92% of all tested patients and 0.68% of COVID+ patients left the ED without a final COVID diagnosis. The amount of time COVID-19 negative patients spent in isolation in the ED decreased by an average of 129.7 ± 3.3 minutes post-POCT implementation.

**Conclusion:**

Use of ED POCT for COVID-19 in the ED resulted in more rapid time to test results and fewer patients leaving the ED who did not know their COVID-19 status, decreasing the likelihood that a COVID+ patient would unknowingly expose others in the community. Additionally, the improved time-to-test-result reduced the amount of time COVID-negative patients spent in isolation, enabling clinical staff to avoid unnecessary PPE usage and free up negative pressure beds.

**Disclosures:**

**Katherine ZJ Fenstermacher, PhD**, Cepheid: Grant/Research Support **Jeanne Mumford, MT**, Cepheid Speaker Bureau: Advisor/Consultant|Cepheid Speaker Bureau: Honoraria **Richard E. Rothman, MD, PhD**, CEPHEID: Advisor/Consultant|Cepheid: Grant/Research Support|Danaher: Grant/Research Support

